# Group A streptococci induce stronger M protein-fibronectin interaction when specific human antibodies are bound

**DOI:** 10.3389/fmicb.2023.1069789

**Published:** 2023-01-26

**Authors:** Sebastian Wrighton, Vibha Kumra Ahnlide, Oscar André, Wael Bahnan, Pontus Nordenfelt

**Affiliations:** Division of Infection Medicine, Department of Clinical Sciences, Faculty of Medicine, Lund University, Lund, Sweden

**Keywords:** antibodies, co-evolution, immune subversion, group A – beta hemolytic streptococcus, fibronectin, adaptive immunity

## Abstract

Group A streptococcus (GAS) is a highly adapted, human-specific pathogen that is known to manipulate the immune system through various mechanisms. GAS’ M protein constitutes a primary target of the immune system due to its spatial configuration and dominance on the bacterial surface. Antibody responses targeting the M protein have been shown to favor the conserved C region. Such antibodies (Abs) circumvent antigenic escape and efficiently bind to various M types. The ability of GAS to bind to fibronectin (Fn), a high molecular weight glycoprotein of the extracellular matrix, has long been known to be essential for the pathogen’s evolutionary success and fitness. However, some strains lack the ability to efficiently bind Fn. Instead, they have been found to additionally bind Fn *via* the A-B domains of their M proteins. Here, we show that human Abs can induce increased Fn-binding affinity in M proteins, likely by enhancing the weak A-B domain binding. We found that this enhanced Fn binding leads to a reduction in Ab-mediated phagocytosis, indicating that this constitutes a GAS immune escape mechanism. We could show that the Fc domain of Abs is necessary to trigger this phenomenon and that Ab flexibility may also play a key role. We, moreover, saw that our Abs could enhance Fn binding in 3 out of 5 emm type strains tested, belonging to different clades, making it likely that this is a more generalizable phenomenon. Together our results suggest a novel synergistic interplay of GAS and host proteins which ultimately benefits the bacterium.

## Introduction

*Streptococcus pyogenes*, also commonly referred to as group A streptococcus (GAS), is an extremely successful, ubiquitously found, human pathogen causing >600 million infections each year. For the most part these infections result in mild disease development such as pharyngitis or pyoderma. However, severe and invasive disease manifestations, including acute rheumatic heart disease, sepsis, and necrotizing fasciitis result in a mortality rate of up to 25% (Carapetis et al., 2005; Walker et al., 2014). Fibronectin (Fn), a high-molecular-weight glycoprotein of the extracellular matrix (ECM), is important due to its ability to serve as an adaptor protein of the ECM, allowing cells to interact with their environment. This is enabled by heterodimeric cell surface receptors known as integrins (Labat-Robert, 2012). Fn exists in an insoluble form whereby it is a constituent of many extracellular matrices and a soluble form that is found in various bodily fluids (Labat-Robert, 2012; Hymes and Klaenhammer, 2016). It is a protein dimer consisting of two almost identical monomers with multiple binding domains (Pankov, 2002). The binding of Fn by GAS is a well-documented phenomenon commonly implicated in enhancing adhesion to and invasion of host cells (Preissner and Chhatwal, 2014; Brouwer et al., 2016; Hymes and Klaenhammer, 2016). This binding is mediated by surface-expressed bacterial proteins, which possess Fn-specific binding domains. Such specialized Fn binding proteins expressed by GAS include PrtF1/SfB1, PrtF2, FbaA, FbaB/PrtF2-like, Serum opacity factor, and SfbX (Henderson et al., 2011). Bound Fn can thus promote host tissue invasion by interacting with integrins expressed on epithelial cells (Cue et al., 2000; Wang et al., 2006; Hauck et al., 2012). As many as 12 GAS surface proteins have been identified as directly or indirectly facilitating the binding of Fn (Yamaguchi et al., 2013). This fact alone hints at an evolutionarily driven process since bacteria would not waste energy on the production of superfluous proteins.

The GAS M protein exemplifies how the bacterium has evolved to manipulate the host immune system. Since the N-terminal domain is highly variable between various GAS serotypes, it has become common practice to classify GAS depending on the specific M protein they express (Frost et al., 2020; Li et al., 2020). M protein dominates the surface of the bacterium and, thus, is likely one of the main targets of the humoral immune response. Despite this, it has been shown that antibody (Ab) responses to M only weakly target the more exposed variable regions and preferentially target the conserved C region (Lannergård et al., 2011, 2015). Due to their conserved nature, these serve as cross-serotype antigens for the adaptive immune system (Metzgar and Zampolli, 2011). However, some M proteins can bind the Fc region of Abs, allowing them to reverse the antibody orientation and reduce immune activation (Nordenfelt et al., 2012). The successful development of a vaccine against GAS has eluded researchers for many years. This has mainly been due to the complex immune response to the M protein. While the conserved regions have been shown to elicit more robust cross-species immune responses, these regions have also been implicated in inducing various autoimmune sequelae (Cunningham, 2014; Good et al., 2015). Moreover, certain M types have been shown to possess the ability to bind Fn and thus increase adhesion or trigger internalization (Ochel et al., 2014; Speziale et al., 2019). Specifically, the M1 protein has been studied in this regard. While the exact binding mechanism remains unclear, it was possible to identify potential Fn-binding regions in the two apical domains (A and B) (Cue et al., 2001).

In recent years, advances in sequencing technology have uncovered a plethora of Fn-binding proteins being expressed by a wide range of bacterial species (Henderson et al., 2011). It is generally accepted that bacteria primarily express proteins that improve or maintain their respective fitness. Since the cost of protein production is so immense, low-benefit proteins are not maintained, and eventually, their encoding genes will be lost altogether (Hottes et al., 2013; Price et al., 2016). This highlights the critical role that Fn-binding must play for GAS fitness and perhaps contributed to its success as a species. M1 GAS has for long periods been observed to be the most prevalent M strain – especially regarding strains implicated in invasive disease (Li et al., 2020). Here, we show that certain M types, including M1, have evolved to exploit human Abs targeting M. The Abs target a conserved and immunodominant region in M protein, making them commonly available to the streptococci. The binding of these Abs allows M protein to bind Fn with high affinity, making additional specialized proteins redundant. The M protein is well known to be versatile. This adds another way in which GAS can use the host’s immune response to its advantage and gain an evolutionary edge.

## Results

### Convalescent patient plasma enhances Fn-binding to GAS

**Figure 1:**
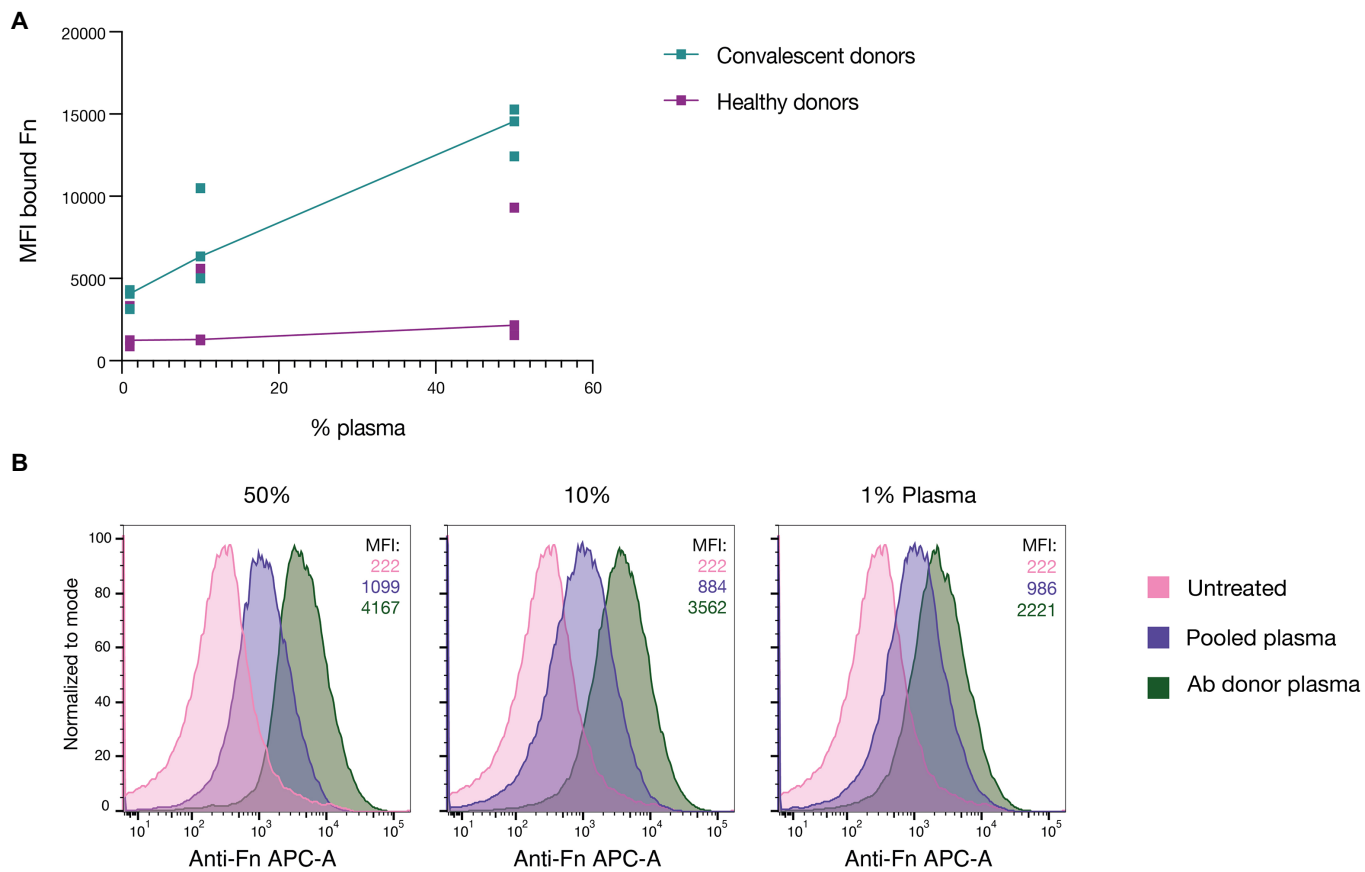
Convalescent patient plasma enhances Fn-binding to GAS. **(A)** GAS was incubated in plasma from patients who had recently recovered from a severe GAS infection (convalescent donors) as well as in plasma from healthy controls. This was done at three different dilutions (50, 10, and 1%). All healthy donor plasma, except one, led to greatly reduced Fn-binding to GAS compared to the convalescent donor plasma. Each point represents a distinct donor, and the line shows the respective median. **(B)** GAS incubated in plasma derived from the original donor of AB25, 32, and 49 led to a dose-dependent increase in bound Fn compared to both untreated bacteria and pooled plasma (MFI of bound Fn displayed in the upper right corner). GAS was incubated in three different plasma dilutions in PBS (50, 10, and 1%). Each histogram corresponds with 20,000 bacterial events as assessed by flow cytometry and they are presented as normalized to mode. All data for this figure was acquired by flow cytometry.

Plasma contains large quantities of Fn (Pankov, 2002), which is present in its various isoforms. It has been shown that the adaptive immune system will target surface-bound bacterial Fn-binding proteins with Abs, leading to better vaccine-provoked protection (Brown et al., 2005; El Ghany et al., 2007). To assess whether GAS infections can lead to *in vivo* generation of Abs that can neutralize Fn-binding proteins, we incubated the M1 serotype SF370, expressing GFP, in various dilutions of plasma derived from convalescent donors who had recently recovered from a severe invasive GAS infection (Figure 1A). As a control, we used plasma from healthy donors. After incubation, the bacteria were washed in PBS, and the bound Fn was stained using an anti-human Fn primary Ab and a fluorescent anti-mouse secondary. The amount of fibronectin bound to GAS was assessed by flow cytometry. Surprisingly, GAS incubated in the convalescent plasma consistently showed higher levels of bound Fn compared to the healthy controls. This was the case for all dilutions, whereby 50% plasma led to the most Fn being bound. This discrepancy was observed between all individuals except one healthy donor where the difference was not as pronounced. To determine whether the same would apply to plasma from a convalescent donor whose blood was used to derive anti-GAS monoclonals (Bahnan et al., 2022) we repeated the assay with plasma from this specific donor (Figure 1B). For this experiment pooled plasma (from >20 healthy individuals) was used as a control which allowed us to better assess the average response of the broader population. Bacteria were left untreated in culture media to assess background autofluorescence. Fn-binding to GAS was measured by flow cytometry. As can be seen from the respective MFI values of the bound Fn, GAS incubated in dilutions of the Ab donor plasma consistently bound more Fn compared to those incubated in pooled plasma. For all dilutions, the untreated GAS and pooled plasma-incubated GAS showed the same binding level, whereas there was a dose-dependent increase when using convalescent donor plasma. In summary, incubating GAS in plasma derived from convalescent patients led to increased Fn-binding compared to when GAS was incubated in plasma from healthy donors. When repeating the assay using plasma from the original convalescent donor – from which our anti-GAS monoclonals are derived – we saw that plasma derived from the Ab donor was also able to promote increased Fn-binding compared to pooled plasma.

### Certain monoclonal Abs induce increased Fn-binding affinity to M1 GAS in an M protein-dependent manner

**Figure 2:**
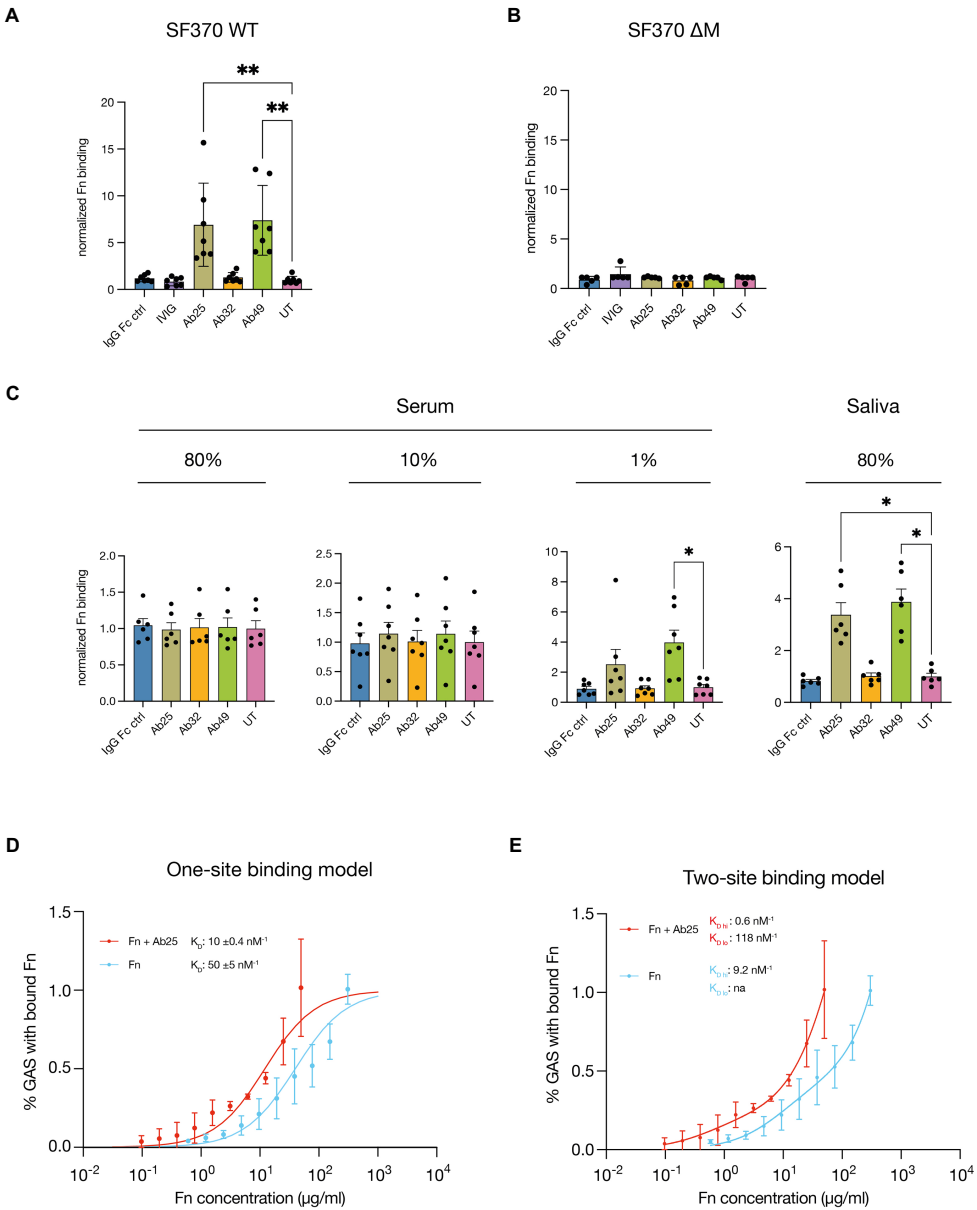
Certain monoclonal Abs induce increased Fn-binding to M1 GAS in an M protein-dependent manner. **(A)** The M-specific monoclonals Ab25 and 49 lead to a significant increase in Fn-binding to SF370 GAS compared to the untreated control (UT, no additional Ab treatment). The Fc-binding control (IgG Fc ctrl), pooled intravenous Abs (IVIG), and the M-specific monoclonal Ab32 did not. **(B)** The M1 protein knock-out mutant SF370 ΔM was treated with Fn alone (UT) or in combination with the Fc control mAb (IgG Fc ctrl), IVIG, and the M-specific monoclonals (Ab25, 32, and 49). All Abs and Fn were added at 20 μg/ml. No significant difference in Fn-binding was observed for any of the treatments compared to the untreated control. **(C)** Lower Ab background levels (80% saliva and 1% serum) result in an increased GAS Fn-binding compared to the untreated control (UT, no additional Ab treatment) when Ab25 and 49 are substituted while this was not seen with other Ab treatments. Higher Ab titers found in 80 and 10% serum reduced the effect substituted Abs had on GAS Fn-binding (all Abs were added at 20 μg/ml). **(D)** Fn binding affinity increases in the presence of Ab25. Binding curves of Fn with and without 20 μg/ml Ab25 give an estimate of affinity to protein M1. Fibronectin binding was measured using flow cytometry. The figure shows measured binding with fitted ideal binding curves as a function of the total fibronectin concentration. *N* = 3 for all concentration points. Dissociation constants (*K*_D_) for the curves are given in the plot, together with a confidence interval calculated using the Bootstrap method. **(E)** A two-site binding model was applied to the same data. High and low estimates for the *K*_D_ values are given in the plot. No *K_D_* value was attainable as a low estimate for Fn alone. Normalization for panels **(A–C)** was performed by dividing the MFI values by the mean MFI of corresponding untreated samples. Each data point for panels **(A–C)** represents the normalized measurement of a bacterial mid-log culture each grown from a distinct colony. Error bars represent the SEM. Statistical significance was assessed using Kruskal–Wallis combined with Dunn’s multiple comparisons test and *denotes *p* ≤ 0.05 and **for *p* ≤ 0.005.

Next, we wanted to assess whether the effects on Fn binding were due to the Abs contained within the respective plasma samples. We therefore incubated SF370 GAS with fluorescently conjugated plasma Fn in conjunction with anti-M protein monoclonal Abs (Figure 2A). The Abs used were the monoclonals (Ab25, Ab32, and Ab49) derived from the aforementioned convalescent donor. We used a monoclonal (human anti-IgE, Xolair) with no specificity for GAS, to control for potential Fc binding mediated effects by the M protein (IgG Fc ctrl). Further, pooled intravenous Abs (IVIG) were used as a positive binding control since we know it contains Abs targeting a wide range of GAS surface proteins (Nordenfelt et al., 2012). GAS-bound fibronectin was assessed by flow cytometry. Incubation with the anti-GAS monoclonals Ab25 and Ab49 both led to a significant increase in Fn-binding to the bacteria while all other Ab treatments did not. To test whether this phenomenon is M protein-dependent we repeated the assay with a M protein knock-out mutant SF370ΔM (Figure 2B). This led to no difference in Fn-binding between the various treatments. To be sure that the Abs themselves were not able to bind Fn, we performed an ELISA with Ab-coated wells. We saw that no Fn was bound to the Abs unless GAS was present (Supplementary Figure S1).

To understand how polyclonal Abs and other serum proteins can influence monoclonal-induced Fn-binding we incubated SF370 in varying concentrations of pooled serum as well as pooled saliva (Figure 2C). Monoclonals were supplemented to the saliva or serum and bound fibronectin was again detected with the anti-Fn monoclonal. GAS Fn binding was assessed by flow cytometry. Fn-binding by GAS incubated in high serum concentrations (80 and 10%) was blunted, and no difference could be seen between the treatments. It is, however, noteworthy that higher serum concentrations led to higher background binding of Fn due to generally higher Fn concentrations. At 1% serum and in saliva, Ab25 and Ab49 treatment consistently resulted in an increased binding of serum Fn by GAS compared to when incubated in serum alone (untreated, UT). Fn-binding was significantly increased for both Ab25 and Ab49 in saliva. In 1% serum, only Ab49 led to a significant increase in Fn-binding.

Since we saw that increased serum concentrations reduced the effect that our monoclonals had on Fn binding we wanted to evaluate whether pooled serum Abs alone could lead to this effect. We therefore incubated SF370 with Fn combined with IVIG (100 μg/ml) and varying concentrations of Ab25 – one of the monoclonals which consistently led to enhanced Fn-binding. We chose this IVIG concentration since it roughly amounts to the average Ab concentration we would expect in 1% serum (Jazayeri et al., 2013). As a control, bacteria were incubated with only IVIG. For normalization, SF370 was left untreated, whereby only Fn was added. The signal of GAS-associated fluorescent Fn was assessed by flow cytometry. GAS treated with Ab25 led to a statistically significant increase in bound fibronectin compared to the Ab untreated control (Supplementary Figure S2). When the same amount of Ab25 was used in conjunction with 100 μg/ml of IVIG there was still an increase in Fn binding albeit a non-significant one. By increasing the amount of Ab25 present together with IVIG, it was possible to outcompete the negating effect of IVIG, again leading to a statistically significant increase in Fn binding (Supplementary Figure S2). Taken together, these results show that both Ab25 and Ab49 can specifically enhance Fn binding on GAS and that the dampening effect of pooled Abs (IVIG) can be outcompeted.

We next wanted to assess the Fn-binding affinity to GAS with and without Ab25. We measured bound Fn at varying concentrations and performed binding curve analysis to determine a relative dissociation constant (*K*_D_) (Figure 2D). Since we could not assume which type of binding would apply, we decided to first use a more conservative one-site model. For the one-site model, we found that while Fn alone bound with a medium-high affinity of 50 ± 5 nM^−1^, the addition of 20 μg/ml of Ab25 led to a binding affinity of *K*_D_ 10 ± 0.4 nM^−1^. This constitutes a fivefold increase in Fn-binding compared to Fn alone. However, at certain concentrations, the binding curve did not seem like a good fit which could indicate that the difference in binding affinities might be underestimated. We, therefore, decided to also apply a two-site binding model (Figure 2E). Here we found a *K*_D hi_ of 9.2 and an unstable *K*_D lo_ for Fn alone. The addition of Ab25 led to a *K*_D hi_ of 0.6 and a *K*_D lo_ of 118. This would mean that the addition of Ab25 could lead to a 19-fold increase in Fn binding affinity. A comparison of the two fit models showed that the two-site model is more likely to be correct. Moreover, by titrating Ab25 in combination with 1 µg/ml of Fn, we found that Ab25 had a half-maximal effective concentration (EC_50_) of 1.1 ± 0.2  nM^−1^ in terms of enhancing Fn binding to GAS (Supplementary Figure S3). In summary, we found that certain monoclonal Abs targeting the M protein are able to induce increased Fn-binding to GAS while non-specific or pooled Abs were not. When these Abs were used in conjunction with saliva or low-concentration plasma, we saw a similar phenotype which was diminished when higher concentrations of plasma were used. We saw that while pooled Abs could dampen the increase in Fn binding induced by our monoclonals, this effect could be outcompeted. Finally, Fn-binding affinity measurements showed that Ab25 allows Fn to bind to the bacterial surface with manyfold increased affinity.

### Ab enhanced Fn binding reduces phagocytic efficiency in monocytes

**Figure 3:**
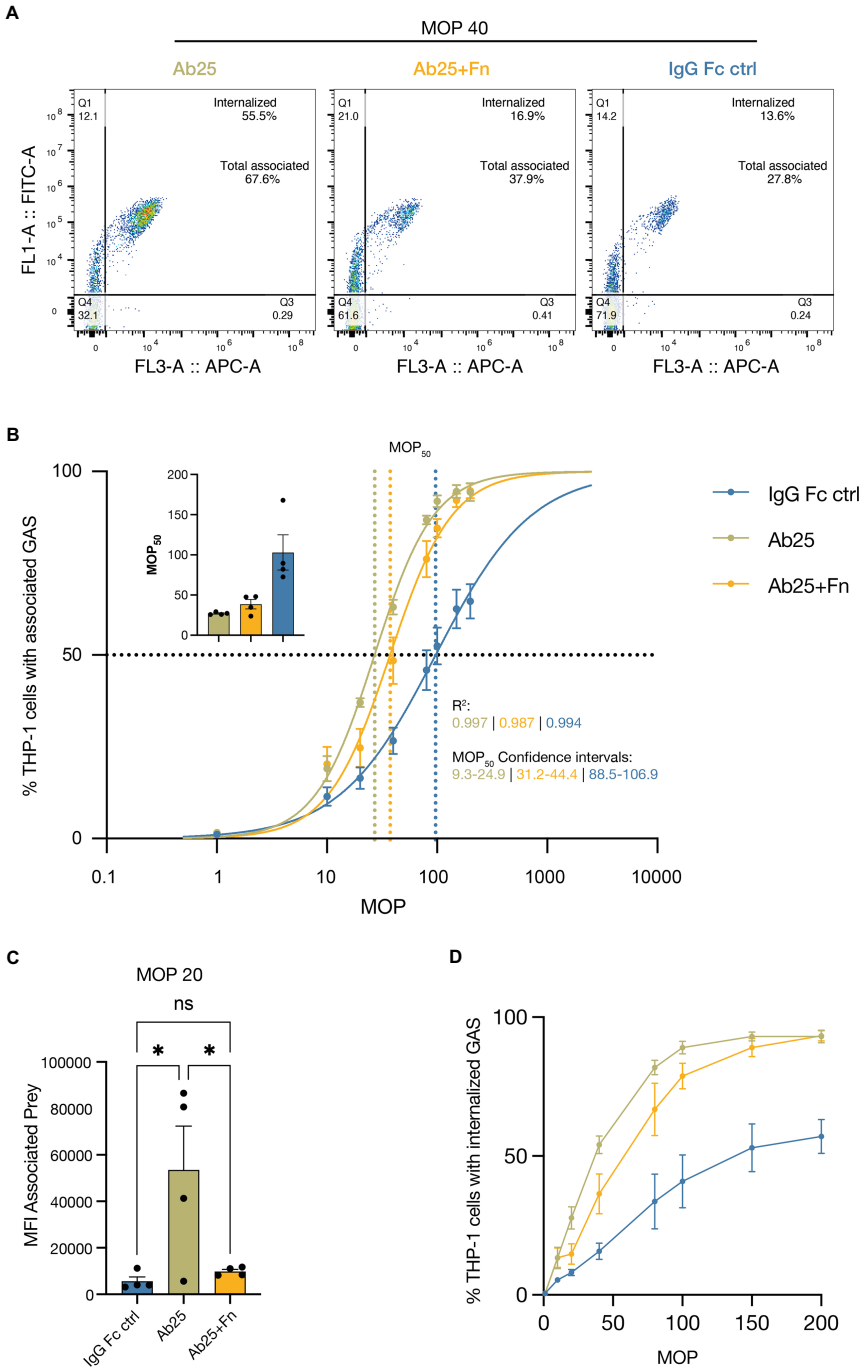
Ab enhanced Fn binding inhibits monocyte phagocytosis. THP-1 cells were incubated with increasing MOPs (multiplicity of prey) of heat-inactivated SF370 bacteria [opsonized with 20 μg/ml of the IgG Fc ctrl, Ab25, or Ab25 + Fn (both 20 μg/ml)]. The THP-1 cells were allowed to associate with and internalize the bacteria for 30 min before flow cytometric analysis. **(A)** Representative flow cytometry plots for each treatment where cells were exposed to opsonized GAS at an MOP of 40. ‘Internalized’ signifies the percentage of cells with internalized prey and ‘total associated’ signifies percentage of cells with both attached and internalized prey. **(B)** The curves represent the percentage of cells associated with bacteria as a function of the MOP. The inset to the top left displays the MOP_50_ for each opsonization condition. *R*^2^ represents the coefficient of determination for the respective treatment curve. MOP_50_ CI represents the 95% confidence interval for the respective MOP_50_ values. **(C)** MFI values of prey (GAS) associated with THP-1 cells when cells were exposed to 20 GAS per FIGURE 3 (Continued)cell (MOP 20). Ab25 led to a significant increase in associated prey compared to both the IgG Fc ctrl and Ab25 + Fn. **(D)** The curves represent the percentage of cells with internalized prey. Statistical significance was assessed using one-way ANOVA combined with Dunnett’s multiple comparisons test and * denotes *p* ≤ 0.05. The curves represent the percentage of cell with internalized prey as a function of the MOP. The presented data represents four independent experiments done on separate days. For panels **(A,D)** the data points represent the mean and the error bars represent the SEM. For panel **(C)** the bars represent the mean, data points represent separate experiments, and the error bars represent the SEM. All data was acquired with flow cytometry.

A key process utilized by the immune system to facilitate pathogen elimination is phagocytosis. We therefore decided to assess the effects of Ab enhanced Fn binding on phagocytic efficiency using a method described in de Neergaard et al. (2021) and chose the well-established monocyte cell line THP-1 as the model phagocyte. The key aspect of this phagocytosis method is to use the ability of phagocytes to associate with their prey as a means to find the right dynamic range to do comparisons between treatments or conditions. It is otherwise a risk to add too few or too many bacteria and not detect potential differences. Both association and internalization are important aspects to consider when evaluating phagocytosis. Ab25 was the obvious candidate to evaluate since it both enhances Fn binding and has, in previous work, been shown to be a powerful opsonin (Bahnan et al., 2022). We incubated the THP-1 cells with varying multiplicities of prey (MOP). An MOP of 10 would mean that for each THP-1 cell we add 10 pre-opsonized bacteria. After 30 min at 37°C, the degree of phagocytosis was assessed by measuring the percentage of THP-1 cells with associated and or internalized prey. This was done with GAS opsonized with either Ab25 or Ab25 + Fn. The IgG Fc ctrl was included as a control to assess the extent of non-opsonic phagocytosis. Figure 3A shows representative flow cytometry plots for a MOP of 40. The flow cytometry gating strategy used for this assay is shown and described in Supplementary Figure S4.

We chose to assess the degrees of persistent association since these have been shown to correlate well with the opsonic capacity of the used Abs and allows us to use non-linear regression analysis to more easily compare the various treatment groups (de Neergaard et al., 2021). The baseline degree of association achieved with a non-opsonic Ab (IgG Fc ctrl) amounted to an average MOP_50_ (number of prey per cell needed to achieve 50% association of THP-1 cells) of 97. Ab25 alone was the most effective at increasing association and exhibited a mean MOP_50_ of 27. Adding Fn to Ab25 resulted in a reduced association efficiency (mean MOP_50_: 37) which can be seen in the graph as a right shift. This means that more bacteria are needed to achieve maximum association. The individual MOP_50_ values for each experimental replicate can be seen in the panel in the upper left corner of the graph. The differences in MOP_50_ were all statistically significant since none of the 95% confidence intervals overlapped (Figure 3B). Next, we assessed the signal of prey associated with cells at a MOP of 20 (20 bacteria per cell). This allowed us to assess any differences in average amount of bacteria associated with THP-1 cells. Here we found a significant difference between Ab25 and Ab25 + Fn as well as Ab25 and the IgG Fc ctrl. There was no statistical difference between the Ab25 + Fn and the IgG Fc ctrl (Figure 3C). By using a pH-sensitive dye, we were able to assess internalization of bacteria. For Ab25 + Fn treated bacteria, we found that consistently fewer THP-1 cells internalized bacteria compared to GAS treated with Ab25 (Figure 3D). Overall, we found that Ab enhanced Fn binding can reduce association and internalization of GAS by monocytes.

### Fn is bound to the upper domain of M1 and Ab flexibility as well as intact Fc domains are necessary for enhanced Fn binding

**Figure 4:**
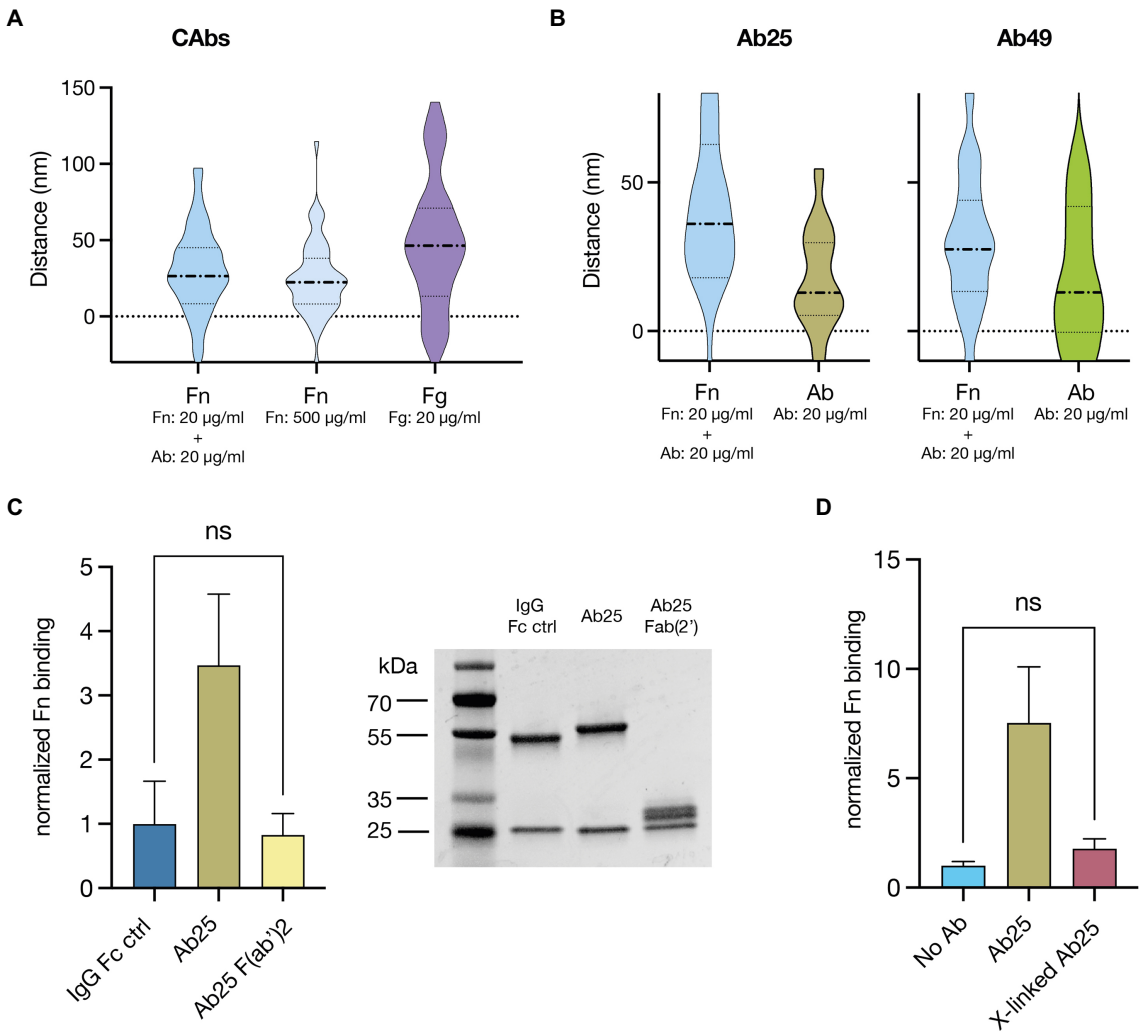
Fn binds to the upper domain in M proteins and Ab flexibility as well as intact Fc domains are necessary for enhanced Fn binding. **(A)** Fn and fibrinogen (Fg) binding site distance measurements from the bacterial surface are displayed as violin plots. Fn-binding resulting from CAb treatment and low-affinity Fn-binding, achieved by incubating the bacteria in high concentrations of Fn are displayed next to the results from a binding distance control Fg. Both Fn-binding measurements showed that Fn consistently bound below Fg on the M protein. **(B)** Violin plots showing binding site distance measurements of Fn and corresponding Ab used to induce said Fn-binding (Ab25, Ab49). Regardless of the Ab treatment, the measured Fn-binding distance was very similar and was in both cases larger than the Ab binding distance. **(C)** Ab25 F(ab’)2 fragments lead to no significant increase in Fn binding compared to the non-binding IgG Fc control (left panel). Full digestion of Ab25 into F(ab’)2 fragments was assessed by SDS page (right panel) **(D)**, Ab25 PFA crosslinked (*x*-linked) on the surface of GAS led to no significant increase in Fn binding compared to the an Ab untreated control.

We wanted to know if the Fn-binding site on the M1 protein is truly in the N-terminal A-B repeats as suggested previously (Cue et al., 2001) and whether this binding site remains the same if Fn-binding is induced by bound Abs. To assess this, we used a microscopy-based binding site localization method (Kumra Ahnlide et al., 2022). This allowed us to measure the distance of a target protein from the cell wall with nanometer-scale precision. Distance measurements with control proteins that bind to known sites on M1 served as landmarks and allowed us to extrapolate which relative region fibronectin was binding to. First, we assessed the Fn-binding induced by isolated convalescent plasma Abs and low-affinity Fn-binding by incubating with a high Fn concentration (500 μg/ml). As a control, we assessed the binding distance of fibrinogen (Fg) (Figure 4A). The measurements showed that both CAb-induced high-affinity Fn-binding and low-affinity binding of highly concentrated Fn led to binding in the same region of M (median binding distance: 26.5 and 22.4 nm respectively). This is below the binding distance of Fg (median binding distance 46.4 nm), which is known to have multiple binding domains in both the A and B repeats and, therefore, also displays a larger data spread (Macheboeuf et al., 2011). Next, we measured the binding distance for Fn bound due to Ab25 or Ab49 and, as controls, assessed the binding distance of the Abs themselves, which have both been shown to bind close to the central S region of M1 (Bahnan et al., 2022; Figure 4B). We found that again, Fn was being bound in a similar location to the previous Fn-binding distance measurements (median binding distance: 35.2 and 27.7 nm). The corresponding Ab binding measurements presented smaller distance values (median binding distance: 11.9 and 13.5 nm), verifying that Fn was binding above both Ab binding epitopes on M protein.

While it was clear that certain Abs are necessary to induce high-affinity Fn-binding to GAS we had not yet assessed how they do so. First, to test the involvement of the Ab Fc domain, we measured Fn binding when GAS is treated with intact Ab25 compared to F(ab)‘2 fragments of the same Ab. As a baseline, we used an Fc-binding IgG control. We found that while Ab25 led to a many-fold increase in Fn binding, the F(ab)‘2 fragments had no significant effect compared to the control (Figure 4C). Next, we wanted to assess the importance of Ab flexibility in regard to Fn binding. To test this, we used paraformaldehyde (PFA) cross-linking. We either fixed GAS before or after opsonization with Ab25. This way, the latter resulted in cross-linked, less flexible Abs on the bacterial surface. The bacteria were thereafter exposed to Fn. We found that while the PFA fixed GAS with regular Ab25 bound a substantial amount of Fn compared to the control, there was no significant difference for GAS with PFA cross-linked Ab25 (Figure 4D).

### Monoclonal Abs induce enhanced Fn-binding in several tested M types

**Figure 5:**
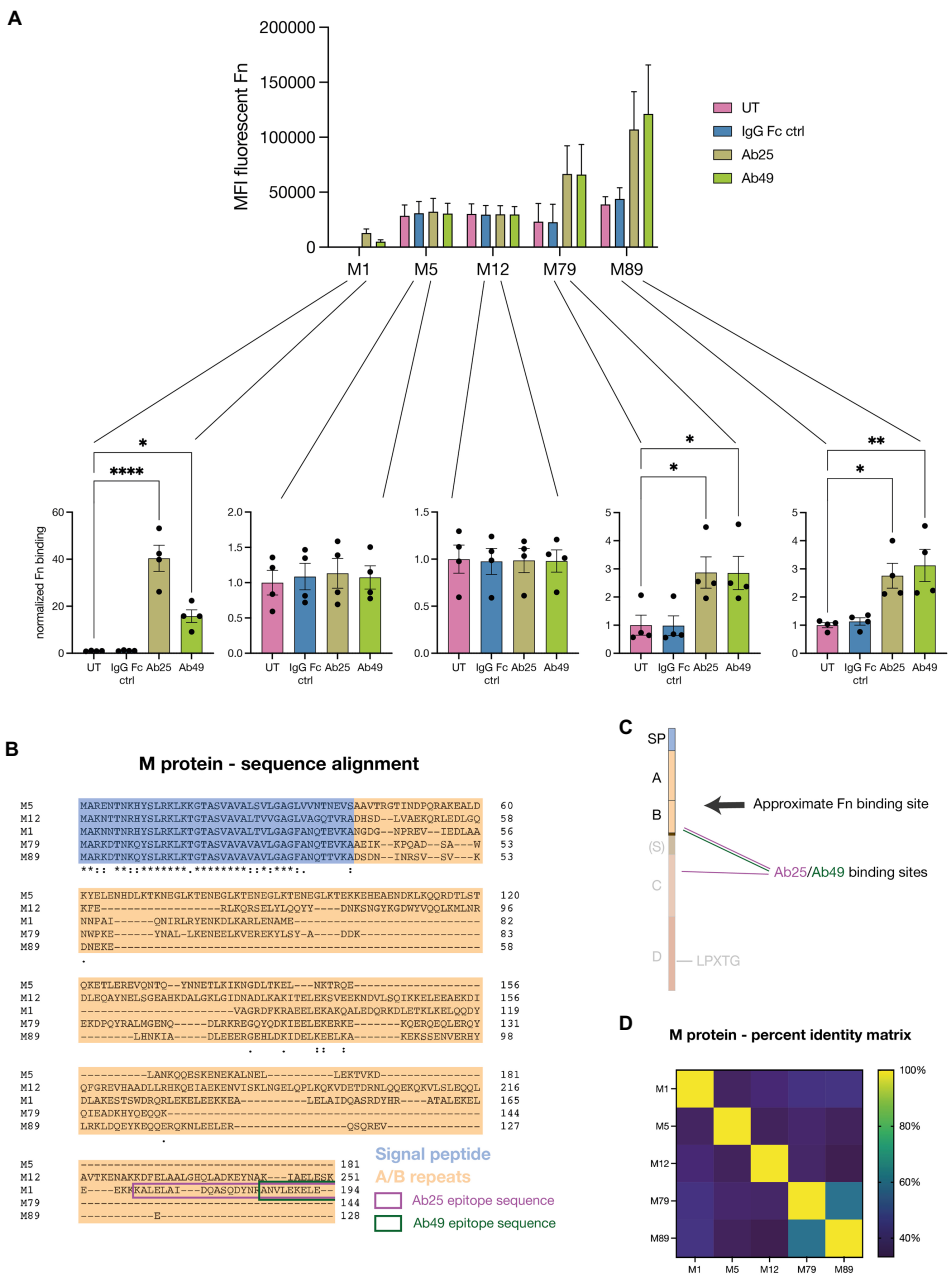
Monoclonal Abs induce enhanced Fn-binding in several tested M types. **(A)** Five GAS strains expressing distinct M types (M1, M5, M12, M79, and M89) were exposed to Fn in combination with Ab treatments. All strains showed variable levels of background Fn-binding when no treatment (UT) or no specific Ab (IgG Fc ctrl) was present. For 3 of the 5 tested strains (M1, M79, and M89), treatment with the monoclonals Ab25 or 49 resulted in a significant increase in Fn-binding compared to the untreated control. All Abs were added at 20 μg/ml. The data for this graph was acquired by flow cytometry and each data point represents the normalized measurement of a bacterial mid-log culture each grown from a distinct colony. **(B)** Amino acid sequence alignment of the various tested M types. The sequences include the signal peptide (blue), A/B regions (orange), and the binding epitope sequences of Ab25 and 49 are framed in magenta and green, respectively. **(C)** Illustration depicting the GAS M protein, divided into its various regions whereby not all M types feature an S region. The binding sites of Ab25 and 49 are shown in magenta and green respectively, whereby Ab25 binds to the M protein with both of its Fab domains in dual-Fab cis binding conformation (Bahnan et al., 2022). Site localization analysis (Figures 4A,B) revealed that Fn binds above both Abs. **(D)** Percent identity matrix showing the similarity of the aligned amino acid sequence of all tested M types. The data is shown as a heatmap. Normalization was performed by dividing the MFI values by the mean MFI of corresponding untreated samples. Error bars represent the SEM. Statistical significance was assessed using one-way ANOVA combined with Dunnett’s multiple comparisons test and *denotes *p* ≤ 0.05, **for *p* ≤ 0.005, ***for *p* ≤ 0.001, and ****for *p* ≤ 0.0001.

We knew from previous work that our Abs broadly react with multiple M types (Bahnan et al., 2022). This presented us with the opportunity to assess how generalizable the described phenomenon is amongst various M strains and if we would see a similar pattern than we had previously seen with Ab binding to GAS. We tested our monoclonal Abs’ ability to induce Fn-binding with a total of five GAS strains expressing various M types (M1, M5, M12, M79, M89) (Figure 5A). We decided to use strains with substantial genetic diversity and therefore chose 2 strains from each M clade. Like M1, M5 and 12 both belong to the Y clade while M79 and M89 belong to the opposite X clade (Sanderson-Smith et al., 2014). We found that compared to the M1 strain, all four other tested strains exhibited far higher background levels of Fn-binding – between ~50 and ~150 times more Fn was bound by untreated (UT = no Ab) GAS strains compared to the M1 strain. The strains M1, M79, and M89 all bound substantially higher amounts of Fn when exposed to our monoclonal Abs (Ab25 and Ab49) while the M5 and M12 strains did not. This matched the pattern seen for Ab binding to the strains (Bahnan et al., 2022). The Fn-binding data were additionally normalized to the UT controls for each strain which allowed for better visualization of the effect each Ab treatment had on each separate strain. We found that, in terms of fold change, the M1 strain was impacted most by the Ab treatment where, on average, Ab25 led to 40-fold more Fn being bound. While we saw a significant increase in Fn-binding for the strains M79 and M89, the high background levels of bound Fn in the UT group made the effect less pronounced.

Next, we wanted to better understand how these distinct M types could be binding Fn under the influence of our bound monoclonals. We, therefore, decided to sequence the various *emm* genes encoding for the M proteins expressed by these strains. Since we had previously determined that Ab-induced Fn-binding occurs in the variable A/B repeats, we aligned the amino acid sequences of all five strains (Figure 5B). To allow for better alignment, we included the signal peptide in the analysis. Our alignment showed that while there were naturally strong homologies within the signal peptide sequence, the A/B repeats showed almost none at all. The binding epitopes of both Ab25 and Ab49 have been framed in magenta and green. For better comprehension, we have included a cartoon of M protein to illustrate the Ab binding sites and proposed relative Fn-binding site (Figure 5C). We used a percent identity matrix to compare sequence similarities between M types (Figure 5D). It shows that even though M1, M79, and M89 all bound increased levels of Fn under the influence of our monoclonals, their variable A/B were not substantially more similar than M5 and M12, which did not. An only slightly higher semblance was observed between M79 and M89. Fn-binding data comparing five different M types showed that different M strains bind vastly different base levels of Fn and that our monoclonals targeting the conserved region of M led to significantly increased binding in 3 of 5 strains. This matched the capacity of the monoclonals to bind to the strains themselves as seen in a previous study (Bahnan et al., 2022). By comparing the amino acid sequences of the variable regions of the various M proteins, we found that the ability to bind Fn to the M protein under the influence of bound Abs did not correlate with substantial sequence homology. This indicates, however, that there at least must be structural homology in the final coiled-coil structure of the M proteins.

## Discussion

It is well known that various GAS strains possess a multitude of Fn-binding proteins which are used to recruit Fn to the bacterial surface – assisting in the process of colonization and infection of a host. We were surprised to find that a GAS strain of the most common M type (M1) bound only insignificant amounts of Fn when incubated in human plasma. We knew that this weak binding was not due to the culture conditions since other M type strains grown in the same way readily bound Fn. Further, we found that incubation in plasma of convalescent patients led to a substantial increase in bound Fn instead of an expected reduction due to Ab interference. It is important to point out that one of the healthy control plasma samples led to a similar increase. While this donor was in good health at the time of donation and could not remember any recent infections, we believe it is possible that they may have unknowingly suffered from a subclinical infection triggering an immune response. However, this would be difficult to assess since essentially all donors will possess some titers of anti-GAS Abs.

Using purified Fn and anti-M monoclonal Abs we found that it was in fact certain M protein targeting Abs that led to this increase in Fn-binding and that pooled Abs from healthy donors (IVIG) led to no such increase. When GAS was incubated with varying concentrations of pooled serum in combination with the anti-M Abs, we saw that higher serum concentrations led to a dampened effect of the Abs on Fn-binding. This was most likely due to anti-GAS polyclonal Abs found in the pooled serum that interfered with the monoclonals’ binding. Serum contains large quantities (10–20 mg/ml) of IgG which means that the 20 μg/ml of our Abs amounts to a very small relative concentration. This would explain why we see that at lower serum concentrations, as well as in saliva, the anti-M Abs had a pronounced effect on Fn-binding. It could also indicate that this phenomenon is a niche-specific effect. This would make sense since GAS most commonly infects and colonizes the nasopharynx – an environment with comparatively low concentrations of IgG. This niche specificity, also reported for Fc binding function in GAS (Nordenfelt et al., 2012), could also explain why we see slight differences between 1% serum and saliva. While these most likely contain comparable Ab concentrations, there are many serum proteins, not found in saliva, which could be interfering in unknown ways. Our data also shows that when it comes to Fn binding to M1, it is not the concentration of Fn that is most important. Rather it is the type of Abs that are present which will affect the amount of Fn bound to M1. We saw that very high concentrations of purified Fn (>500 μg/ml, Figure 3B) will bind to M1 spontaneously – typical for a low affinity interaction. However, this was not the case for pooled plasma which also contains high concentrations of Fn. We believe that this is due to the high concentrations of interfering anti-GAS Abs found there. This hypothesis is supported by the data shown in Supplementary Figure S2. There we show that pooled intravenous Abs (IVIG), previously shown to readily bind to and opsonize GAS (Nordenfelt et al., 2009), did not affect, or even slightly reduced, Fn binding to GAS. We, moreover, show that far lower concentrations of our anti-M monoclonals can outcompete this binding and trigger significantly increased Fn binding. This indicates that a small Ab subpopulation is sufficient to induce this phenomenon. In fact, we found that one of our three anti-M monoclonals, Ab32, did not lead to such an effect. This could be due to this Ab’s binding epitope – since Ab25 and 49 essentially share a binding epitope. It is possible that this phenomenon only occurs when Abs bind to a very specific region of the M protein.

We at first assessed Fn binding affinity with a one-site binding model. This revealed that Fn binding affinity could be increased fivefold by the addition of Ab25. However, since it was clear that the binding curve was an imperfect fit at certain Fn concentrations we decided to also apply a two-site binding model. This showed that Fn binding affinity is increased around 19-fold in the presence of Ab25. We have decided to include both binding models since we do not wish to make assumptions regarding the specific binding mechanics. However, the fit for the two-site model does seem like a superior fit to the data. Moreover, previous findings do indeed indicate that Fn binding occurs on two distinct areas of the M1 protein (Cue et al., 2001). And yet, this does not serve as definitive evidence of two-site binding. It is possible that the Ab binding to the M protein only affects one of the sites. Further, the structural complexity of the M protein makes it hard to rely on classical protein manipulation techniques such as those used in the aforementioned study.

To test the biological relevance of Ab enhanced Fn binding we used a flow cytometry-based phagocytosis assay. We assessed three distinct parameters which are either indirect or direct indicators of efficient phagocytosis (percent of phagocyte-prey association, fluorescent signal from phagocyte associated prey, and percent of phagocytes with internalized prey). Linear regression analysis showed that the addition of Fn reduced the number of monocytes that were associated with GAS at a certain MOP – indicating reduced opsonic efficiency. This also translated to significantly fewer bacteria being associated with monocytes on average. Moreover, by staining the bacteria with a pH sensitive dye we were able to assess how many monocytes contained internalized GAS. Overall, we found that adding Fn to the opsonin Ab25 reduced phagocytic efficiency in the monocyte cell line. This Ab targets the M protein C repeats which have been seen to act as an immunogenic flag, actively drawing in an Ab response (Lannergård et al., 2011, 2015). It has not fully been understood why GAS would benefit from actively encouraging the generation of such Abs since some can be detrimental to the bacteria (Bahnan et al., 2022). However, these results may give some insight into a mechanism by which the effects of such effective opsonic Abs can be blunted by exploiting another ubiquitously found host protein.

While these effects on phagocytosis are intriguing it will be critical to further study the physiological implications caused by this phenomenon. GAS has already been linked to many post-infection autoimmune sequelae such as rheumatic carditis, glomerulonephritis, and Sydenham chorea. All these diseases are mediated by Abs which are thought to be generated due to molecular mimicry between the M protein constant regions and various human tissues (Cunningham, 2016). Moreover, it would be important to understand how it is beneficial for the bacteria to possess a protein with a function which can be activated through outside influence. It is possible that this phenomenon acts as a sensing mechanism – triggering accumulation of Fn on the bacterial surface just when the immune system begins mounting an immune response. This could potentially allow the bacteria to ‘react’ to a forthcoming hostile immune response. This would not be the first report of GAS exhibiting mechanisms to ‘sense’ environmental processes. Previous observations found that by employing the major GAS surface protein SclA/Scl1, GAS can adapt and respond to the host’s wound environment by selectively binding wound associated isotypes of Fn (McNitt et al., 2019). Future studies exploring the physiological impacts of Ab enhanced binding of Fn to M protein could prove essential in better understanding the highly complex relationship between GAS and the immune system.

**Figure 6:**
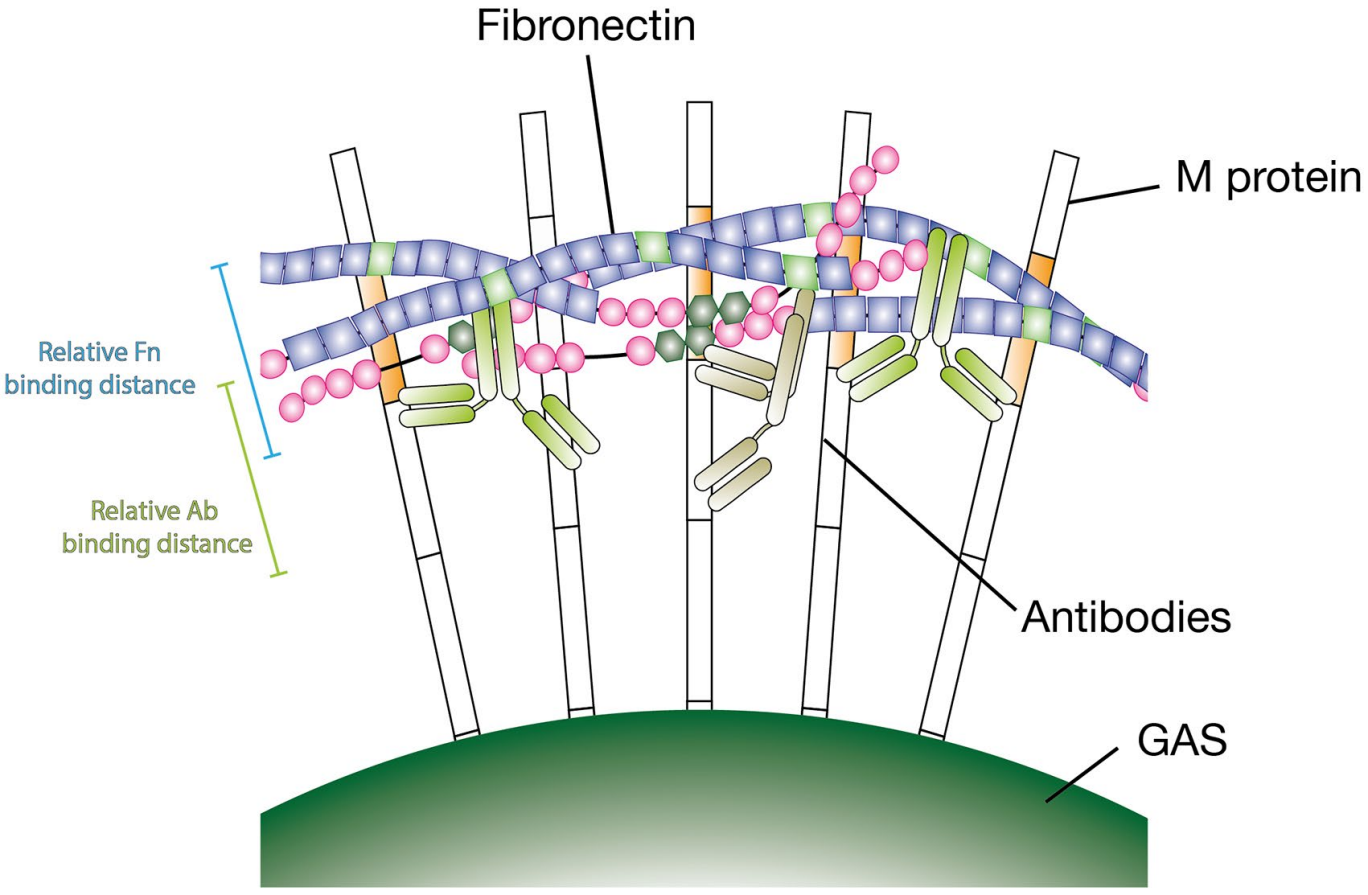
Hypothetical model of antibodies inducing Fn-binding to M protein. Illustration showing a hypothetical binding model of Abs inducing Fn-binding to surface-bound M protein. Specific antibodies are required to bind at a particular distance from the bacterial surface. Intact Ab Fc domains are required to enhance Fn binding. Flexible antibodies increase likeliness of Fc domains being able to interact with Fn. The scale bars represent relative binding distances attained from binding distance measurements (Figure 4B).

The M protein is a structurally complex nonideal α-helical coiled coil (Ghosh, 2011). The nonideal pairing of the protein dimers’ amino acid sequences makes the M protein’s specific conformation highly volatile and influenced by environmental factors such as temperature and pH alterations. This has substantial effects on its function (Akerstrom et al., 1992; Nilson et al., 1995). In fact, a closely related M-like protein, protein H, has been shown to synergistically bind C4BP through Fc bound IgG. The authors suggest that binding of C4BP is enhanced due to the Fc bound Abs stabilizing protein H in its dimeric conformation (Ermert et al., 2019). This could partly explain our findings although it is important to point out that here it is specific Fab binding Abs enhancing the Fn binding. Further, we found that the non-GAS-bound Ab Fc domains play a different important role. We tested F(ab’)2 fragments of Ab25 in our GAS Fn binding assay and found that only intact Ab25 Abs were able to enhance Fn binding. Moreover, to assess the importance of Ab flexibility we tested the effect of PFA crosslinking which is known to make proteins more rigid. Here again we saw that modifying the Abs resulted in a near complete loss of function. Only unfixed Ab25 led to an increase in Fn binding compared to the no Ab control. Both these findings offer us a better insight into the mechanisms at play. We deem it likely that the bound Abs act as a type of protein scaffold. Our findings show that Fn binds in the A-B repeats – just above the bindings site of both Ab25 and 49. It is possible that an interaction between the Ab Fc domains and Fn must occur in order to enhance Fn binding. The fact that more rigid crosslinked Abs lose functionality would also support this theory since the Fc of flexible Abs would more easily be able to bend upwards toward the bound Fn. This hypothetical binding mechanism is shown in Figure 6. While we have explored many aspects of Ab enhanced M protein-mediated Fn-binding the exact mechanism remains unknown. Further in-depth studies will be necessary to fully understand this phenomenon.

Finally, since we knew that the Fn-binding was occurring in the variable N-terminal region of M we were curious if our monoclonals targeting the central region would induce the same response in other M types (Bahnan et al., 2022). We found that, of the four additionally tested M types, two bound more Fn in the presence of the monoclonals. It was the M79 and M89 strains from the opposite M clade which did so (Sanderson-Smith et al., 2014). Amino acid sequence analysis revealed that the variable A-B repeats of all five strains differed significantly showing no clear sequence homologies. This indicates that, as previously shown for M-like proteins, it is the complex dimeric quaternary structure that determines their function as well as the binding of Abs to them (Frick et al., 1992; Nilson et al., 1995; Ermert et al., 2019; Hauri et al., 2019; Bahnan et al., 2022). However, it is more difficult to understand the phenomenon’s usefulness for these strains since they already express highly efficient Fn-binding proteins. It does, however, explain why the Fn-binding function of these two M proteins seems to have been overlooked up until now.

*Streptococcus pyogenes* was one of the first bacterial pathogens proven to bind the host protein Fn to its surface. It quickly became clear that this capability must be key to stable colonization of a host and that it benefits the bacterium (Henderson et al., 2011). Upon more in-depth study, highly complex immune-subversive mechanisms between GAS-bound Fn and host cells came into focus (Talay et al., 1992; Cue et al., 1998; Yamaguchi et al., 2013). In recent years many proteins responsible for Fn-binding have been discovered and, in fact, it is often the case that GAS strains express multiple heterologous Fn-binding proteins simultaneously (Hymes and Klaenhammer, 2016). Protein production is a costly process in terms of energy consumption and so, from an evolutionary standpoint, it makes no sense for bacteria to express proteins that do not benefit them. This makes it clear that Fn-binding must have been a crucial function during the millennia of co-evolution with the human immune system. We found that SF370, the M1 strain used in this study, was only able to bind Fn very weakly. This is despite the fact that its genome contains the known Fn-binding protein FbaA (Ferretti et al., 2001) and even its M protein has been shown to be able to bind Fn (Cue et al., 1998, 2001). We must therefore ask ourselves: what is the benefit of these Fn-binding proteins if they result in such weak Fn binding? The findings detailed in this study indicate that certain M1 GAS strains can more efficiently bind Fn when anti-M Abs are present. Is it possible that M1 strains are partly so successful since this mechanism gives them an evolutionary advantage?

It would be interesting to further assess how Ab-enhanced Fn binding affects the virulence of GAS. Previous work found that complementing an M1-type strain with the Fn-binding protein F1 resulted in attenuated virulence *in vivo* (Nyberg et al., 2004). At first, this may seem contradictory since GAS-mediated Fn-binding has so clearly been linked to virulence (Talay et al., 1992; Yamaguchi et al., 2013; Singh et al., 2015; Speziale et al., 2019). However, this may be an indication of the importance of Ab enhanced M protein-mediated Fn-binding. If certain M1 strains have adapted to be highly reliant on a more energy-efficient means of binding Fn, such as binding Fn with the assistance of binding Abs, then the forced expression of a superfluous protein could lead to metabolic dysregulation resulting in lower fitness and thus virulence. This could also be the case for the investigated M1 type strain SF370. Could evolutionary reliance on Ab-mediated Fn-binding to the M1 protein have led to a low expression of this specialized Fn-binding protein? Further studies including more M1 strains, in particular clinical isolates, will be important to understand whether this is a more generalizable phenomenon among GAS strains. We, moreover, believe that it is possible that human antibodies are being exploited by other bacterial species through similar mechanisms since a wide range of bacterial pathogens express multifunctional coiled-coil proteins on their surface (Hernandez Alvarez et al., 2019). Due to the complexity of such a binding mechanism – requiring the cooperation of three distinct binding partners – we believe it is possible that many such interactions have, until now, remained undiscovered. This again highlights the importance of using a wide variety of methods to test specific questions.

In conclusion, we show a novel immune escape mechanism of GAS involving a complex interplay between host and pathogen-derived proteins. We show that when certain human antibodies bind to the M1 protein, they enhance the binding affinity of the Fn binding region found within the A/B regions. For this to happen, the antibodies require intact Fc domains and likely must be flexible. This enhanced Fn binding due to antibodies protects the bacteria by reducing the likelihood of being cleared by opsonophagocytosis.

## Materials and methods

### Bacterial strains, growth, staining, and transformation

Five different GAS strains, expressing distinct M types were used for this study (M1, M5, M12, M79, M89). Both M1 and M5 expressing strains, SF370 and Manfredo respectively, have been thoroughly studied in previous studies (Ferretti et al., 2001; Holden et al., 2007). The other strains (M12, M79, M89) were clinical isolates derived from routine care patients with severe GAS infections. These were isolated, identified, and emm typed at the Clinical Microbiology Laboratory, Skåne University Hospital, Lund, Sweden. The GAS strains were grown statically in Todd-Hewitt Yeast media (THY) at 37°C, 5% CO_2_. The strains were maintained on THY agar plates for 3 weeks before being replaced with a plate freshly streaked from −80°C stocks. For binding experiments, bacteria were grown to logarithmic phase by diluting an overnight culture 1:20 in fresh THY. After dilution bacteria were grown until they were in the mid-log phase – around 2–2.5 h or until they reached an OD600 of 0.4. For the transformation of GFP expressing SF370 and the M1 knock-out mutant strain ΔM were washed in ice-cold water to make them electrocompetent. These bacteria were electroporated with 20 μg of the pGFP1 plasmid and after a 1-h recovery period, the bacteria were plated on THY plates supplemented with erythromycin. Successful transformants were tested for fluorescence under ultraviolet light. Heat-inactivation of the bacteria was done by growing them to mid-log as described previously. Thereafter, they were washed in PBS before being transferred to ice for 15 min. Next, the bacteria were heat-shocked at 80°C for 5 min and immediately transferred back to ice for another 15 min. For the phagocytosis assay, heat inactivated bacteria were first stained with 5 μ Oregon Green 488-X succinimidyl ester (ThermoFisher) at 37°C under gentle rotation whilst protected from light for 30 min. The bacteria were then centrifuged, washed once in sodium carbonate buffer (0.1 M, pH 9.0) before being resuspended in said buffer to allow for a final staining with the pH sensitive dye CypHer5E (Fisher Scientific). The dye was added at a concentration of 17 μg/ml before a 30-min incubation at 37°C. Thereafter, the bacteria were washed and resuspended in Na-medium (5.6 mM glucose, 127 mM NaCl, 10.8 mM KCl, 2.4 mM KH2PO4, 1.6 mM MgSO4, 10 mM HEPES, 1.8 mM CaCl2; pH adjusted to 7.3 with NaOH). Stained bacteria were stored at 4°C until use. For site localization analysis SF370 GAS was grown to mid-log as previously described. The culture was then centrifuged and washed in 10 ml of PBS. The bacteria were then resuspended in 1 ml of PBS. The bacterial cell wall was, depending on the experiment, either stained with AlexaFluor (AF) 488 or AF594 conjugated WGA. After staining the bacteria were sonicated and then fixed in 1% PFA for 30 min at RT. After fixing, Tris was added for a final concentration of 333 mM to quench PFA before centrifugation. This reduced bacterial clumping during centrifugation.

### Plasma and serum samples

The convalescent patient samples used in Figure 1A were derived from patients who had recovered from GAS bacteremia between 2018 and 2020. This was done after obtaining oral and written consent. Serum was collected after 4–6 weeks after hospital admission. The healthy donor plasma for this panel was taken from 3 healthy donors who had no symptoms at the time of venous puncture. For Figure 1B plasma was derived from the GAS convalescent donor whose b-cells were originally isolated to attain the monoclonals tested in this study (Ab25, 32, 49). The process from discovery to generation of these monoclonal Abs is described in Bahnan et al. (2022). All blood samples were collected with sodium citrate as an anticoagulant after venous puncture. The pooled plasma was commercially available pooled control plasma (Affinity Biologicals Inc.). For the experiments depicted in Figure 2C the pooled serum was purchased from Sigma. The pooled saliva was made by pooling saliva from 10 healthy donors. The saliva was then centrifuged to remove coarse debris and then passed through a 0.2 μm syringe filter.

### Ethics statement

The study with convalescent GAS bacteriemia patients was approved by the regional ethics committee with the permit number 2016/939, amendment 2018/828. The collection and analysis of the other blood samples, including the convalescent donor depicted in Figure 1B as well as the healthy donors, was approved by the regional ethics committee, permit number 2015/801.

### Monoclonal Abs

The monoclonal Abs employed in this study were previously generated and investigated at length (Bahnan et al., 2022). All Abs were of the subclass IgG1. Ab25, Ab32, and Ab49 were found to bind to GAS M protein with high specificity and affinity. The Abs were also shown cross-react with various M types. Cross-linking combined with mass spectrometry revealed that the binding epitopes of all three monoclonals lay close to the S region of the M1 protein. Ab25 presented a special binding conformation whereby it bound the M protein in *cis* dual-Fab conformation. This means that it bound M1 with both its Fab domains simultaneously with epitopes on both sides of the S domain. As a non-binding IgG Fc control Ab, we used the commercially available monoclonal Xolair (Omalizumab, Novartis).

### Antibody purification, digestion, and assessment

Monoclonal Ab generation and purification was done as previously described (Bahnan et al., 2022). Ab purification from plasma was done by incubating plasma with protein G Sepharose beads (Cytiva). The beads were incubated with the plasma for 2 h before being transferred to a chromatography column. The beads were then washed four times with 10 ml of PBS without allowing the beads to dry out between or after washes. The Abs were then eluted with 0.1 M glycine which was immediately buffered with Tris. Centrifugal filter columns (Millipore, Merk) were used to exchange the buffer to PBS. F(ab’)2 fragments of Ab25 were made by incubating 300 μg of the Ab with 10 μg of the enzyme IdeS (Genovis) and Ab cleavage was assessed by SDS page. For Ab25 fixation experiments the protocol for GAS fixation as described above was employed. This was done either before or after opsonization with Ab25.

### Fibronectin and Ab conjugation

Fn purified from human plasma (sigma) or monoclonal Abs were fluorescently conjugated using AlexaFluor 647 Ester (Invitrogen). The conjugation and purification were done according to the manufacturer’s guidelines. Protein concentration after purification, as well as the degree of labeling, was assessed using a DeNovix DS-11 FX system.

### Fn-binding assays and flow cytometry

GFP expressing SF370 WT and ΔM, as well as M5, M12, M79, M89 GAS were grown to mid-log as previously described. The bacteria were centrifuged and thoroughly resuspended in 1 ml of PBS. For patient plasma incubation experiments (Figures 1A,B) varying dilutions of plasma were prepared in a 96-well plate whereupon 10 μl of the mid-log culture concentrate was directly added. As pooled plasma, we used frozen normal control plasma (VisuCon-F, Affinity Biologicals) which, according to the manufacturer, was collected from at least 20 healthy donors. Bacteria were incubated with the plasma for 30 min at 37°C. After incubation, the plate was centrifuged, and the bacteria were washed twice in PBS. The bacteria were stained with an anti-Fn Ab (Thermo Fisher) and incubated for 30 min at 37°C. The bacteria were then washed once to remove the primary Ab and then stained with an anti-mouse fluorescent secondary conjugated with AlexaFluor 647 (Thermo Fisher). Fibronectin binding was assessed by flow cytometry (CytoFlex, Beckman Coulter). GFP-expressing bacteria were assessed by gating for FITC positive events. This gate was then assessed for Fn median fluorescence intensity (MFI) in the APC channel. Since it was not possible to transform all M strains for the cross-strain binding experiment the bacteria were gated using SSC and FSC. The accuracy of this gating strategy was confirmed by comparing both gating methods with GFP-expressing strains. The pooled serum and saliva experiment (Figure 2C) was done following a similar protocol. Pooled human serum (Sigma) dilutions and pooled saliva were prepared in a 96-well plate. The anti-GAS monoclonals Ab25, Ab32, and Ab49 as well as the non-binding IgG Fc control was added at 20 μg/ml before 10 μl of the concentrated mid-log SF370 culture was added. As an untreated control (UT), PBS was added instead of monoclonal Ab treatment. The rest of the experiment was done following the same protocol as mentioned previously. After the acquisition, the MFIs were normalized to the results attained from the untreated samples. The Fn binding experiments only employing purified Abs (Figures 2, 3, 4B–D) were done by preparing a plate with the corresponding Abs (20 μg/ml) and AlexaFluor 647-Fn (20 μg/ml). As an untreated control (UT), only PBS was used. Eighty μl of a non-concentrated mid-log culture of the corresponding bacteria was added to each well. The bacteria were incubated for 30 min at 37°C. After incubation, the bacteria were washed twice with PBS before assessment of bound Fn by flow cytometry as detailed above. The Fn binding ELISA (Supplementary Figure S1) was done by coating ELISA plates overnight with 10 μg/ml of the corresponding Abs in 2% BSA in PBST at 4°C. The untreated (UT) and no Fn wells were coated only with 2% BSA in PBST. For the SF370 + Ab25 control well heat inactivated SF370 in 2% BSA in PBST was used for coating. The wells were washed 3 times with PBST and blocked with 2% BSA in 300 μl PBST for 30 min. After blocking, 100 μl of 10 μg/ml Fn (Sigma-Aldrich) diluted in PBST was added to the wells except the no Fn control well. The samples were incubated for 1 h at 37°C, washed, and 100 μl of a mouse anti-human Fn monoclonal (Thermo Fisher) was used diluted 1:1000 in 2% BSA in PBST. The plate was incubated 1 h followed by 3 washes. Finally, 100 μl/well of a goat anti-mouse IgG-HRP (Novus Biologicals) diluted 1:3000 in 2%BSA was added to the wells and incubated at 37°C for 1 h. The samples were then washed and developed with 100 μl developing reagent (20 ml Substrate buffer NaCitrate pH 4.5 + 1 ml ABTS Peroxide substrate +0.4 ml H2O2). Absorbance was read at OD450 following 5–30 min of color development at room temperature.

### Affinity measurements

A 10 ml SF370 culture was concentrated into 1,000 μl of PBS, and 10 μl of bacteria were added to three 1.5 ml tubes – one for each binding curve. Three hundred μg/ml of AlexaFluor 647-conjugated fibronectin was added to the first tube. Fifty μg/ml of AlexaFluor 647-conjugated fibronectin and 20 μg/ml of Ab25 were added to the second tube. One μg/ml of AlexaFluor 647-conjugated fibronectin and 20 μg/ml of Ab25 were added to the third tube. All tubes were incubated on shake at 4°C for 30 min. Bacteria and all constant protein concentrations for each binding curve were added to wells of a 96-well plate – 20 μg/ml of Ab25 in each well for the second binding curve and 20 μg/ml fibronectin in each well for the third binding curve. Serial dilutions were made with the prepared wells using the tube samples as maximum concentration points. The well plate was incubated on shake for 30 min at 4°C. Flow cytometric acquisition was performed using a CytoFLEX. The one-site binding assessment of the theoretical fit was performed in MATLAB using a weighted least squares method for an ideal binding curve with the dissociation constant as an unknown variable. The accuracy of predicted affinity estimates was calculated using the Bootstrap method (Efron, 1979) and is the confidence interval calculated from 50 resamplings of the acquired data. The two-site binding assessment was done in GraphPad Prism by using the non-linear regression analysis for two-site specific binding.

### SIM imaging

For the determination of the Fn-binding site, the bacteria were prepared as mentioned above. The site localization experiments were done with SF370, whereby the cell wall was stained with AlexaFluor 488-conjugated wheat germ agglutinin (WGA). They were then opsonized with AlexaFluor 647-conjugated Fn and corresponding non fluorescently labeled Abs. All treatments were used at 20 μg/ml except high Fn, used to show low-affinity Fn-binding, for which 500 μg/ml of Fn was used. To ensure thorough opsonization the bacteria were incubated with their corresponding treatments for 30 min at 37°C while shaking. After incubation, the bacteria were washed once in PBS. Samples were mounted on glass slides using Prolong Gold Antifade Mountant (Invitrogen) with 1.5H coverslips. Single bacteria were manually identified and for site localization determination they were imaged with a time series of 15 images per channel. Images of single bacteria were acquired using a Nikon N-SIM microscope equipped with a LU-NV laser unit, CFI SR HP Apochromat TIRF 100X Oil objective (N.A. 1.49), and an additional 1.5x magnification. The camera used was ORCA-Flash 4.0 sCMOS camera (Hamamatsu Photonics K.K.). Reconstruction was done with Nikon’s proprietary SIM software included in NIS Elements Ar (NIS-A 6D and N-SIM Analysis). The analysis pipeline for site determination was implemented in Julia and is available on GitHub (Kumra Ahnlide et al., 2022). Representative illustrations showing hypothetical protein binding to M were made in Adobe Illustrator.

### Phagocytosis assays

Phagocytosis assays were performed by employing persistent association normalization (de Neergaard et al., 2021). Pre-stained heat-inactivated GAS were sonicated for 6 min (VialTweeter, Hielscher) to disperse bacterial aggregates. Sufficient sonication was confirmed by microscopy. The bacteria were quantified by flow cytometry. The pH responsiveness of the CypHer5E stain was tested by assessing bacterial fluorescence in the APC channel before and after the addition of sodium acetate (3 M, pH 5.0). Bacteria were opsonized with 20 μg/ml of the IgG Fc control monoclonal (Xolair), the anti-M monoclonal Ab25, and purified serum Fn (Sigma) for 30 min at 37°C in Na medium. To assess multiple MOPs, a 96-well plate was prepared with corresponding dilutions of pre-opsonized bacteria whereupon a set number of THP-1 cells (100,000) was added to each well. Cells were allowed to associate with the bacteria for 30 min before being put on ice and kept on ice throughout data acquisition to slow phagocytosis. The samples were assessed by flow cytometry on a CytoFlex flow cytometer. By first gating for single cells in the leukocyte population we were able to assess which cells were associated with bacteria (FITC positive) and those which had also phagocytosed prey (FITC and APC positive). Gating strategy shown in Supplementary Figure S4. Non-linear regression analysis depicted in Figure 3B was done in GraphPad Prism 9 according to methods described in the de Neergaard et al. (2021).

### M strain sequencing and amino acid alignment

The genomic DNA of the 5 assessed M strains were isolated and purified using the Wizard Genomic DNA Purification Kit (Promega). The growing of strains, bacterial lysis, and DNA were done according to the manufacturer’s guidelines. After isolation, the DNA was sequenced using the TruSeq DNA PCR-Free platform from Illumina. The M protein amino acid sequences were aligned and analyzed using Clustal Omega (1.2.4).

### Graphs and statistical analysis

All graphs and statistical analysis were done in GraphPad Prism (9.3.1.). To assess statistical differences in the Fn-binding to SF370 GAS induced by various anti-M monoclonals (Figures 2A–C) we employed the Kruskal–Wallis *H* test combined with the Dunn multiple comparisons *post hoc* test. For the assessment of statistical differences in other experiments we employed an ANOVA combined with Dunnett’s *post hoc* multiple comparisons test.

## Data availability statement

The original contributions presented in the study are included in the article/Supplementary material, further inquiries can be directed to the corresponding author.

## Ethics statement

The studies involving human participants were reviewed and approved by Etikprövningsmyndigheten, Sweden. The patients/participants provided their written informed consent to participate in this study.

## Author contributions

SW, WB, and PN conceptualization. SW, VK, and OA experimentation and data analysis. SW and PN writing original draft. All authors contributed to reading and editing the final manuscript.

## Funding

SW, VK, OA, and WB were funded by the Royal Physiographic Society. PN was funded by the Alfred Österlund Foundation, the Knut and Alice Wallenberg Foundation (KAW) and the Swedish Research Council (VR).

## Conflict of interest

The authors declare that the research was conducted in the absence of any commercial or financial relationships that could be construed as a potential conflict of interest.

## Publisher’s note

All claims expressed in this article are solely those of the authors and do not necessarily represent those of their affiliated organizations, or those of the publisher, the editors and the reviewers. Any product that may be evaluated in this article, or claim that may be made by its manufacturer, is not guaranteed or endorsed by the publisher.
