# Supplementary material for: Group A streptococci induce stronger M protein-fibronectin interaction when specific human antibodies are bound
Source: Front Microbiol. 2023 Jan 26;14:1069789. doi: 10.3389/fmicb.2023.1069789 (PMC9909010; doi:10.3389/fmicb.2023.1069789)
Supplement: Supplementary file 1 [file Data_Sheet_1.PDF]

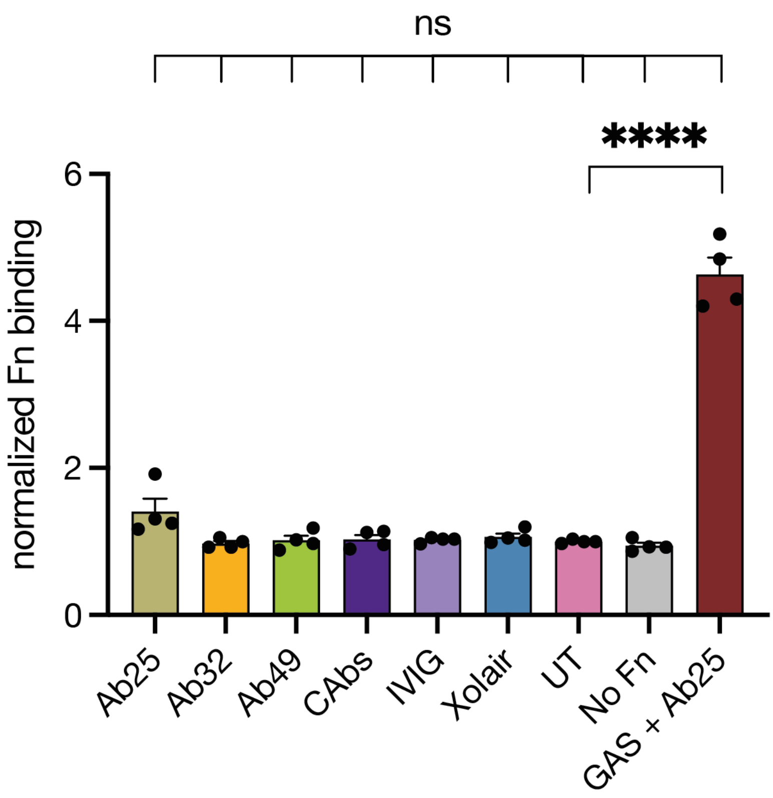

Supplementary Figure 1 Wells coated with various antibodies (10 µg/ml) did not lead to significant binding of Fn (10 µg/ml) compared to an untreated control (UT, no Ab coating). Wells coated with GAS and then treated with the same concentration of Ab (GAS + Ab25) did lead to a multiple-fold increase in Fn-binding. Error bars represent the SEM. Statistical significance was assessed using one-way ANOVA followed by Dunnett's multiple comparisons test and **** denotes $p < 0.0001$.

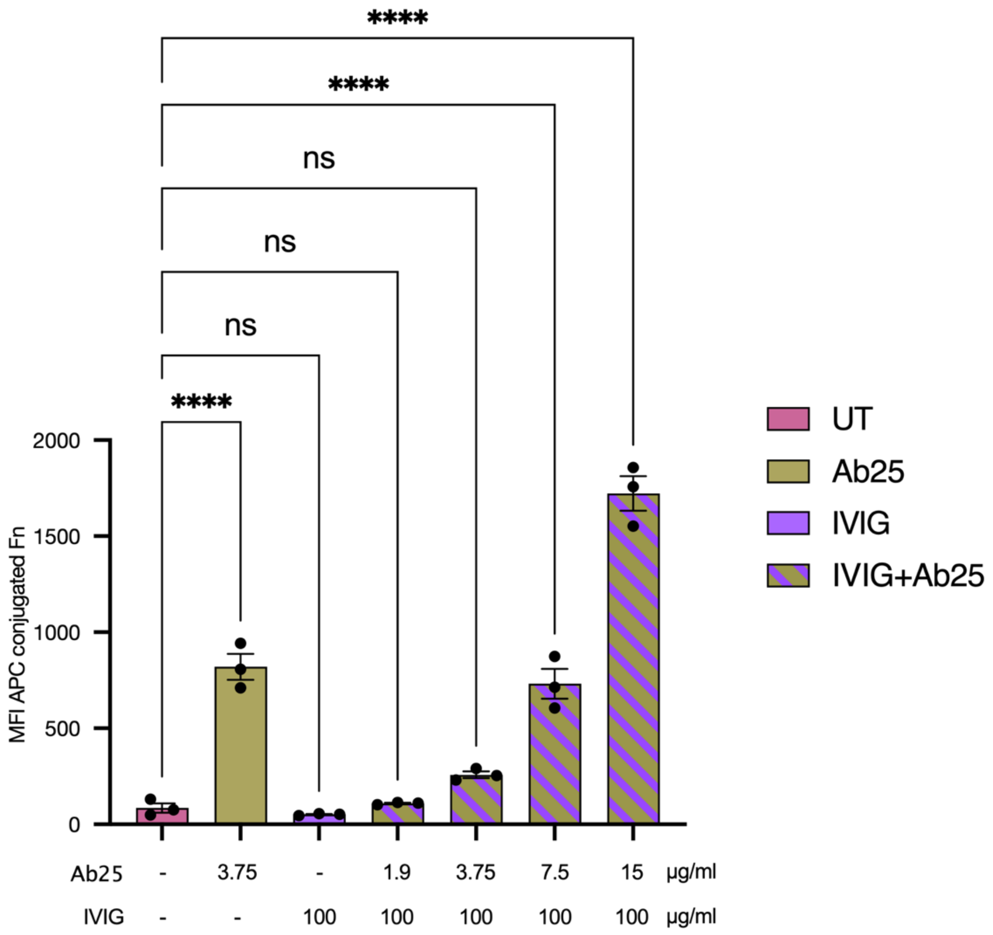

Supplementary Figure 2 Highly concentrated IVIG (100 µg/ml) can be outcompeted by Ab25 (varying concentrations (µg/ml), see graph). All data for this figure was acquired by flow cytometry. The median fluorescence intensity (MFI) of AF647 conjugated Fn bound to GAS was assessed and is shown on the Y-axis. MFI of GAS-bound Fn due to respective Ab treatments was compared to antibody untreated GAS. Each data point signifies the result from a separate experiment. The bars show the mean Fn signal, and the error bars the SEM. Statistical significance was assessed using one-way ANOVA followed by Dunnett's multiple comparisons test ns denotes non-significant and **** for $p < 0.0001$.

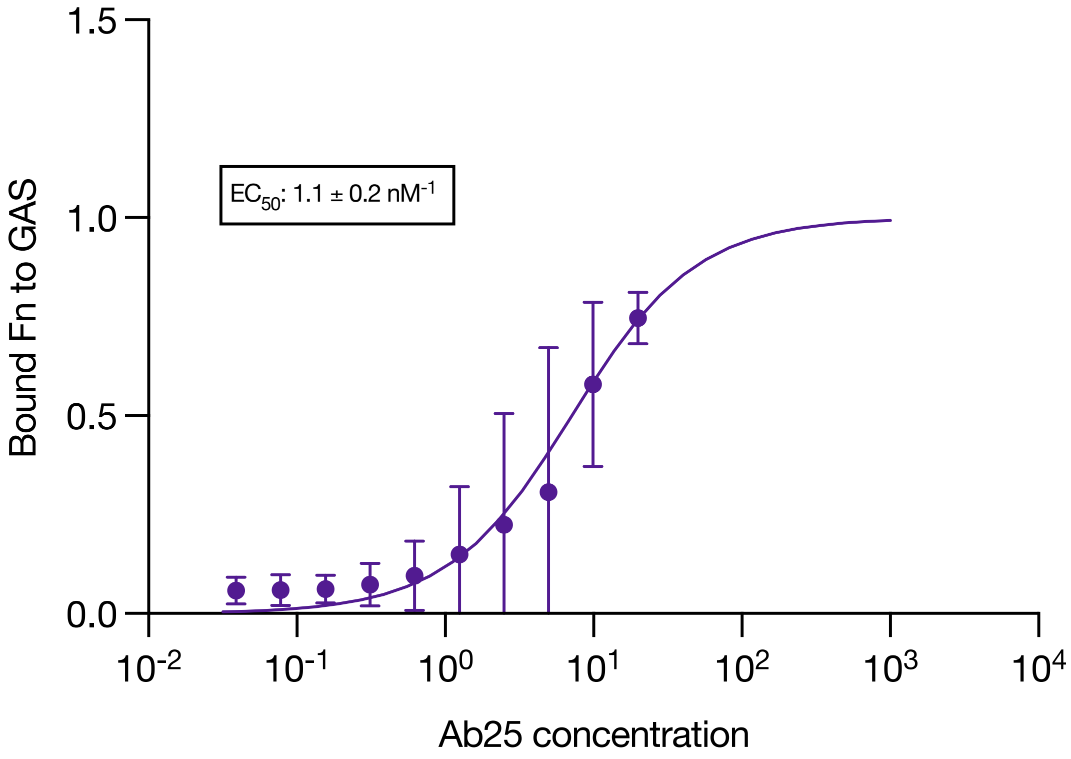

Supplementary Figure 3 Titration of Ab25 shows positive modulation of fibronectin binding. Fibronectin binding was measured using flow cytometry. The figure shows the measured binding of 1 ug/ml of fibronectin with a fitted ideal binding curve as a function of Ab25 concentration. N=3 for all concentration points. The half-maximal effective concentration (EC_{50}) of Ab25 on fibronectin binding is given in the plot, together with a confidence interval calculated using the Bootstrap method.

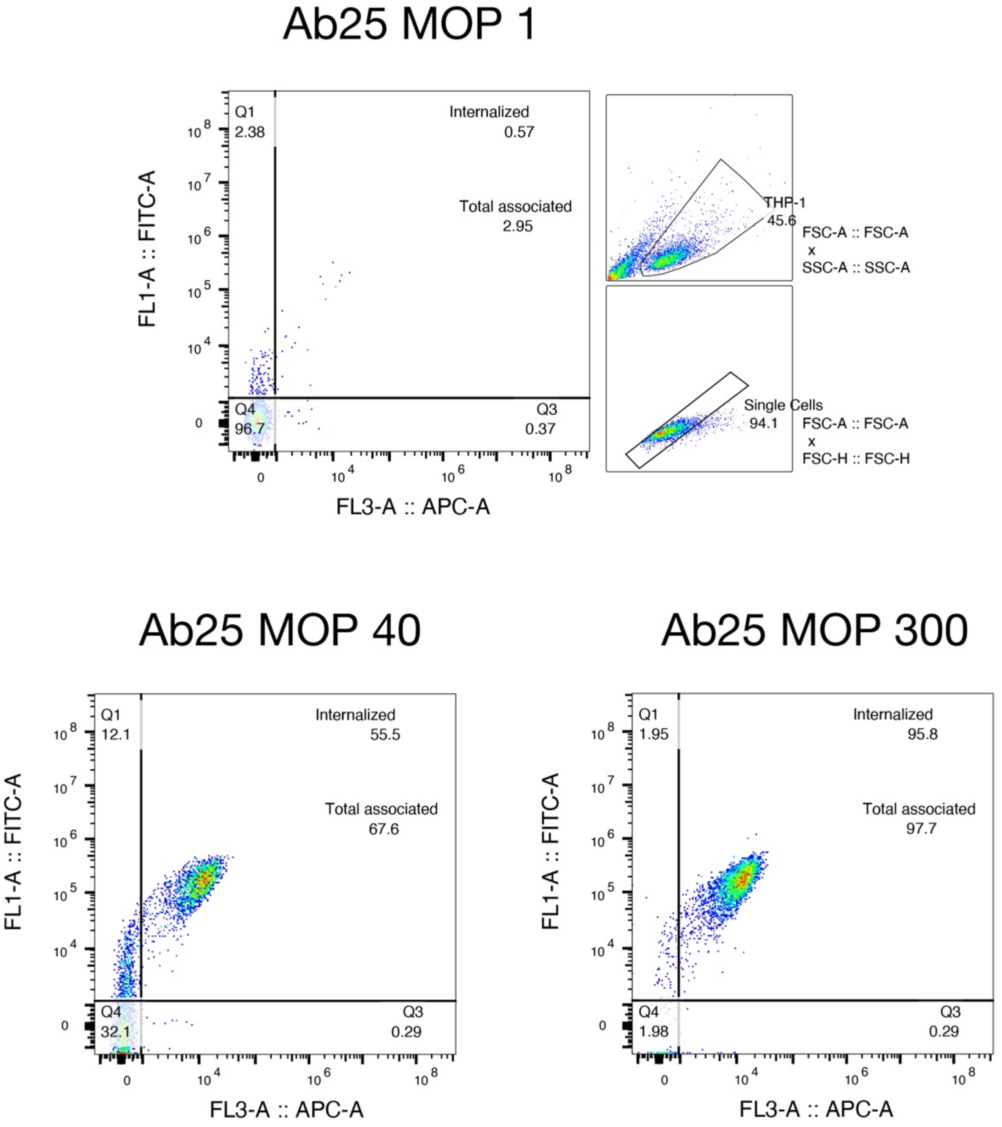

Supplementary Figure 4. Gating strategy for the phagocytosis assay in Figure 5. First, THP-1 cells were gated for on forward- and side-scatter FSC-A x SSC-A. Doublets were excluded through height versus area gating on FSC. Next, an assessment was done on APC-A and FITC-A in order to detect the Oregon green-stained bacteria, which were additionally stained with the pH-sensitive dye CypHer5E. Using a sample with no bacterial treatment, a quadrant gate was drawn around the single-cell population so that FITC-A and APC-A negative cells were in the lower left quadrant (Q4). This way, the upper left quadrant (Q1) signified cells with associated, non-internalized bacteria. And the upper right quadrant (Q2) signified cells with internalized bacteria. Finally, a square gate was drawn around Q1 and Q2 to assess all cells with associated bacteria (associated + internalized).
